# Supplementary material for: Acute effects of kinesiology tape tension on soleus muscle h-reflex modulations during lying and standing postures
Source: PLoS One. 2020 Jul 30;15(7):e0236587. doi: 10.1371/journal.pone.0236587 (PMC7392227; doi:10.1371/journal.pone.0236587)
Supplement: Attachment — Submitted filename: Response to Reviewers.docx [file pone.0236587.s003.docx]

Responses to Comments from Reviewers

**Manuscript Number:** PONE-D-20-05949

**Title:** “Acute Effects of Kinesiology Tape Tension on Soleus Muscle H-reflex Modulations during Lying and Standing Postures”

Summary: We would like to take the opportunity to thank the editor and the reviewer for the comments and suggestions. Following are the comments from the reviewers, as well as our point-by-point responses (*red, italic*) to the comments. In addition, we have revised our manuscript main text based on the comments from the reviewers. Specifically, the revised parts in the new manuscript are track-changed.

Reviewer #1: INTRODUCTION
Lines 54-55: I disagree with this information, as there are several studies demonstrating that, in fact, kinesiotaping does not alter the proprioceptive responses or has a minimally effect. I suggest that you present the current evidence and present the gaps that justify your study. For instance, there are at least two studies using H-reflex measurements within the KT context, showing that the tape does not influence the motoneuron excitability:
<https://journals.humankinetics.com/view/journals/jsr/aop/article-10.1123-jsr.2018->0435/article-10.1123-jsr.2018-0435.xml
https://pubmed.ncbi.nlm.nih.gov/21079436/

*Response: Thank you for the suggestion. We revised the sentences according to your comment, Line 57-65 (in the track-change version). The findings of two references suggested by the reviewer were added in the introduction.*

Lines 58-59: You should further discuss the issue of KT tension and how it is applied, because there are several studies showing wide variability. This raises the issue of a poor reliability regarding the tape tension. In addition, I recommend that you discuss the issue of tape direction, which supposedly influences the motoneuron excitability (by facilitation or inhibition). This is an important aspect that is still controversial.

*Response: Thanks for bringing this point up. We now have added a sentence at the end of the first paragraph: “**The discrepancy of research findings may be due to various methods of KT application, such as the tape tension and the tape direction among different studies.”*

*In addition, we have also strengthened the introduction based on your comment by elaborating the different methods used in kinesiotaping. Please see our track-changes in the Introduction section (Lines 66-71; as well as Lines 102-114).*

METHOD
Please explain in detail the familiarization procedure

*Response: Thanks for this comment. We added detail regarding the familiarization, Line 145-147. “The procedure included introduction of the experiment, familiarization of the H-reflex test, experimental procedures, and determination of physical characteristics.”*

The sample size calculation has missing information. For instance, what was the statistical model adopted for the calculation? Also, what was the effect size considered? And what was the outcome used for the analysis?

*Response: We appreciate this comment. In experimental approach to the problem, Line 153-160. We revised the information for sample size estimation.*

If the participants were physically active and adopted a regular physical exercise routine, how did you manage to discriminate between the effects of their daily routine and the effects of the elastic tape? What kind of exercise they performed? You should include this information as a characterization.

*Response: Thanks for bring this up. The discrimination between the participants daily exercise routine and the effects of the kinesiology tape was reported in the Subjects section, line 174-175. “Participants were required to refrain from any strenuous activity 24 hours before participation.” In addition, the type of exercise they performed was added to limitation section, P20, line 415-416. “They were weekly involved recreational basketball training, amateur takedown training, running, cycling or resistance training.”.*

The post taping h-reflex assessment was performed after the removal of the tape? In this case, how did you manage to control the taping effects in the skin (a possible cutaneous stimulation), from the assessment of the h-reflex?

*Response: The kinesiology conditions to assess the H-reflex response was referred to a previous study (Firth et al., 2010. Clin J Sport Med). In our study, the physical therapist removed the KT taping after the Mid H-reflex assessment. The post KT condition was set to examine the time effect of KT application to the spinal excitability when different stretch tensions were applied.* *To avoid the strong stimuli of cutaneous sensory inputs, we* *carefully removed the tape in a tender manner. These details were added to the contents (Lines 233-234).*

Regarding the tape tension: the procedure is not clear. For instance, how did you manage to control the tension-length ratio, in order to avoid the fracture point? Also, if you reached the maximum stretch intensity, how did you guarantee that this intensity did not compromised the elastic properties of the tape? Several studies adopted equations to control this aspect, by using a percentage of tape tension, in order to reproduce the original instructions of the method… please clarify.

*Response: We provided more information regarding the procedure of tape application, Line 223-229, 237-239, and 246-249.*

*Regarding the compromising elastic properties of the tape, we appreciate your point, and it’s a valid point. We cannot guarantee the similar recoil of the KT tensions throughout the experiment due to a large variant of resistance force as observed at maximal stretch of the tape (see figure 1). We agree this consideration could be a potential limitation of the study.*

It is not clear what was the purpose of the VAS scale. I fail to see any justification to investigate the participant’s perception. Please clarify.
*Response: We appreciate this point. The main reason we had this measurement is that in practicality, many individuals receive the kinesiology tape intervention/treatment, and they perceive the tape tension and they feel “good and effective” of this treatment. However, our study’s findings show the discrepancy between the perception and reality. This is the only purpose/intention of having this measurement. We agree with you that this has nothing to do with the motoneuron excitability, but we feel it can be practically important to keep this information here. If the reviewer still thinks it’s not appropriate having the information here, we’ll be happy to remove such information as well as its results portion.*

*We added information to justify the purpose to measure VAS of tape tensions, Line 276-277. “The purpose of the VAS measurement was to determine the subjective perception of tape tensions of KT during different stretch conditions in lying and standing positions.”*

Statistical analysis:

Lines 243-245: this information is confusing. Why did you adopt t tests?

*Response: Thanks for this comment. We remove paired sample t-tests.*

Cohen’s d should be used in this case, but only the eta partial squared. Cohen’s d is used for bivariate comparisons, which is not the case. Please correct accordingly.

*Response: We fully agree with your suggestion. We removed the sentence “In addition, Cohen’s d was calculated, with 0.2, 0.5, and 0.8 as the thresholds for small, medium, and large effect sizes, respectively.”*

RESULTS
Overall, the results section does not read well. I would recommend to be more objective within the textual presentation of the results, keeping the numbers to a minimum. Details of effect size, mean differences, and so on, should be presented in tables. This would improve the reading.

Please include mean differences and confidence interval of 95% of comparisons.

Table 1 is confusing because it presents the comparisons, but not the data.

*Response: Thank you for the suggestions. Now we have reorganized the results after the VAS part, and added the mean differences and 95CI to Table 1. In addition, the F-values were removed.*

DISCUSSION Lines 301-305: the main findings present a causal relationship, for instance, that posture plays a significant role influencing h-reflex parameters. However, in order to reach this conclusion a parallel design would have been more adequate, and not a repeated measure design with the same participants.

*Response: Thank you for this comment. We agree that a parallel design could have been good for our research question. However, it was not our intention to present a causal relationship. Especially, the posture (standing vs. lying) orders were counterbalance randomized, so were the tension intensities. For example, in the original manuscript, we mentioned “The postures were randomly sequenced at the beginning of each experimental visit, and the sequence was maintained during that specific visit. After a specific posture, the transition to the next posture took approximately 30 seconds, and the participants were asked to maintain the next posture for at least one minute before the H-reflex assessment.”*

*To avoid any further misunderstanding, we have also slightly changed the very beginning part of the Discussion: “The main findings of this study include: 1) the H-reflex parameters (raw H-reflex amplitude and Hmax/Mmax ratio) were significantly lower during the standing posture, when compared to the lying posture, regardless the different tension intensities or measurement time points, and 2) the tape tension factor did not influence the H-reflex modulation.” (Lines 356-360).*

Again, what was the purpose of the VAS? This result does not add to the study, as the authors might have included in order to show that “something happened” and the participants perceived the tension. However, this might bias the readers, as your focus was the motoneuron excitability, and not subject’s perception.

*Response: Thank you for the question. It is indeed, not our intention to bias the readers. As we mentioned in the previous comment: “The purpose of VAS measurement was to determine the subjective perception of tape tensions of KT during different stretch conditions in lying and standing positions.” And here, we emphasize that “Anecdotally, individuals who receive the kinesiology tape intervention/treatment may perceive the strong tape tension as “good and effective” of this treatment.” Line 364-365.*

Lines 351-352: this information is biased, as several studies investigated tape tension and tape direction.

*Response: Thank you for bringing this point. We now have changed the sentence to “Even though the current study had some interesting findings, this experiment did have some limitations.”*

Lines 368-371: there are several studies that already investigated the KT effects within functional performance. This should be discussed in light of your findings.

*Response: Thank you for bringing this point. We have now deleted the sentence regarding the functional performance.*
