# Supplementary material for: Acute effects of kinesiology tape tension on soleus muscle h-reflex modulations during lying and standing postures
Source: PLoS One. 2020 Jul 30;15(7):e0236587. doi: 10.1371/journal.pone.0236587 (PMC7392227; doi:10.1371/journal.pone.0236587)
Supplement: Attachment — Submitted filename: Response to Reviewers PONE-D-20-05949R2.docx [file pone.0236587.s004.docx]

Responses to Comments from Reviewers

**Manuscript Number:** PONE-D-20-05949R2

**Title:** “Acute Effects of Kinesiology Tape Tension on Soleus Muscle H-reflex Modulations during Lying and Standing Postures”

Summary: We would like to take the opportunity to thank the editor and the reviewer for providing the comments and suggestions again. Following are the comments from the reviewers, as well as our point-by-point responses (*red, italic*) to the comments. The revised parts in the new manuscript are track-changed.

Editor: The Reviewer points to significant remaining issues about the sample size calculations and EMG standardization procedures between sessions. The Reviewer also insists that the results and discussion should be focused on the physiological (H-reflex) rather than subjective measures. After examining the manuscript, I have to agree with the Reviewer on most points. I am particularly concerned by the statistical approach, which seems inappropriate. Why a 'repeated measures ANOVA' for post-test, where you should have used a Tukey's unless you used a multivariate approach. In the latter case, you need to describe it clearly in the text. Can you provide a clear description of which variables (Hmax, Mmax, H/M ratio, VAS) were entered in the ANOVA and whether assumptions were met (Levene's test)? Finally, I am concerned by the values of Mmax reported in your manuscript (about 2.5 mV, p.14v, line 314), which are much lower than those reported Mmax for the Soleus (> 10 mV). Providing examples of H and M recording traces between conditions could help resolve the issues. In the next round, please make sure that all concerns, problems and suggestions are fully addressed.

*Response: Thank you again for your detailed comments. We have now made further revision to the manuscript, according to your suggestion, as well as the Reviewer 1’s. We hope the revised manuscript reaches the publication criteria.*

*Specifically, the subjective measure (VAS) has been removed. And the revised discussion is now focused purely on the physiologic observation (h-reflex parameters).*

*In terms of the statistical approach, dependent variables H-threshold, Hmax, Mmax, and H/M ratio were entered in the ANOVA tests. Also, per Reviewer 1’s request, VAS was removed. Briefly, the 3-way ANOVA tests did not show any 3-way or 2-way interactions for all dependent variables. All DVs had significant main effect for posture, and the Mmax also have significant main effect for tension. You are correct that we should not be using 'repeated measures ANOVA' for post-test. We performed pairwise comparison (Bonferroni adjusted) for postures for each DV, and for tension for Mmax. However, we are not sure if Levene's test is the proper test to test the assumptions of equality of variances, because there are no between-subject factors. Instead, we have included Mauchly's Test of Sphericity. These details have been provided in the revised Results section (Lines 324-327, revised manuscript with track-changes).*

*For the Mmax value, our raw data shows a narrow range of 2.81mV-3.07 mV in lying condition and 2.38 mV-2.75 mV in standing condition. Our previous study showed a repeated measure of mean value of 3.29 mV and 3.5 mV (Chen et al., 2010, J Electromyogr Kinsiol) during rest in healthy adults. Despite, we used similar technique to identify descending limb of H-reflex and M-wave responses with our previous study, the smaller Mmax value in the present study may be related to different data acquisition system and EMG sensors. Thus, we are providing* *examples of H and M recording traces (see the new Figure 2) between conditions to give readers a better picture.*

Reviewer #1: Dear authors, the current version of the manuscript was improved. However, I still have several comments that might help to improve the quality of your paper. Please consider my suggestions presented below.

*Thank you again for reviewing our manuscript, and the detailed comments. We have now addressed your comments as below, we have also revised the manuscript based on your suggestions.*

Specific comments:
INTRODUCTION
• Line 68: instead of ‘preferred length’, perhaps ‘a specific length that might represent a desired tape tension’ would be more adequate here.
• Line 69: please change ‘may’ to ‘allegedly’, as the mechanisms of KT are not fully proved by research.
• Line 74: please erase ‘muscle fascia and somatic elements’ and change to ‘neuronal excitability due to a tactile stimulus’ (this is the premise of KT).
• Line 74-75: the use of a term such as ‘gold standard’ is mistaken. That would imply an established validity and reliability, which is not the case. At most, you should use something like a ‘lack of standardization’ regarding tape tension.
• Line 125: change ‘can’ to ‘might’.
• Lines 127-129: I suggest removing this last sentence.
• Line 136: erase the term ‘gold standard’, as this is not adequate nor applies to this context. Nevertheless, I suggest removing the last sentences (lines 135-138).

*Response: Thank you very much for the suggestions on the Introduction section. We now have fully taken your suggestions, and revised the manuscript accordingly.*

METHOD
• Thanks for the inclusion of the familiarization procedure. However, please be more specific on how and what was adopted as familiarization?

*Response: Thank you for this comment. We now added more specific information about the familiarization: “Specifically, this visit started with measuring the subjects’ physical characteristics (e.g., standing height, body weight). Following these measurements, the subjects were them familiarized with the H-reflex test, which mainly included surface EMG electrodes placements as well as locating the optimal sites of the* *stimulation electrodes. Lastly, the subjects were asked to practise two postures (lying and standing), during which the experimental procedures were explained.” We hope this description adds more specific information to the familiarization visit (Lines 134-139, revised manuscript with track changes).*

• The sample size calculation description improved; however, it is not clear why you used ‘gait velocity’ as your outcome of interest? This is not adequate, as you should include a suitable variable, for instance, measures of h-reflex based on previous studies. Also, your design would suggest a F test (ANOVA), and not a t test (two dependent means), as you have 3 repeated measures (0, 50 and 100KT).

*Response: Thanks for your comment about this issue. We agree with you, and we have revised the power analysis part based on your suggestion. Due to there was no direct study comparing H-reflex among different KT tape tension, we have only taken the healthy group participants results from Firth et al. (2010) “The effect of kinesiotape on function, pain, and motoneuronal excitability in healthy people and people with Achilles tendinopathy” into consideration. The facilitation effect (healthy group) observed in Firth et al. (2010) was d = 0.55 (moderate), with the moderate tape tension.*

*Thus, we then conducted the A priori power analysis using the G-power (F test ANOVA, repeated measures, within factors). On the basis of the possible large effect size (f = 0.40) for*

*the difference in tape tension, an α level of 0.05, and a power (1-β) of 0.80, it was shown*

*that at least 12 participants were necessary.*

*We now have included the following into the power analysis in the main text: “Based on a previous study conducted by Firth et al. (2010),* *moderate KT application had a moderate effect in facilitating motoneuronal excitability in healthy people. Thus, we conducted the A priori power analysis using the G*Power software (G*Power version 3.1.9.4, Düsseldorf, Germany), on the basis of the possible large effect size (f = 0.40) for the difference in tape tension, an α level of 0.05, and a power (1-β) of 0.80. It was shown that at least 12 participants were necessary (F-test ANOVA, repeated measures, within factors).” (Lines 147-152).*

• Please include more information regarding the EMG acquisition: common mode rejection ration, signal to noise ratio, input impedance, and electrode descriptions (if active or not, and distance between poles). Also, what were the procedures adopted before coupling the electrodes, to improve the signal acquisition and to reduce skin impedance? How did you guarantee the reliability of the EMG electrode placement, considering the different visits to the laboratory? Please clarify.

*Response: Thanks for your comment about this issue. We added additional description to highlight this point.”* *The EMG sensors are active electrodes contained two stainless steel disks of 11.4 mm diameter with a 20-mm interdisk distance. The EMG signal acquisition was processed with common mode rejection ratio of 95 dB, signal to noise ratio > 89 dB, and input impedance of 100 MΩ.” (Lines 193-196)*

*As for the EMG electrodes placement, the electrode placement was marked with indelible ink to ensure same electrode placement among visits. The participants were also asked to remark the placement between consecutive visits (Lines 188-189).*

• Line 237: what do you mean by “facilitated KT application”? Do you mean that the application followed the facilitation technique proposed by the KT method? This is not clear. Also, what process can be used to avoid bias? This information is lost.

*Response: We apologize for this misunderstanding. Here “facilitate” simply meant “conduct/apply”. To avoid confusion, we revised to the sentence to “The intensity of the tape was determined by the length-tension relationship of the kinesiology tape, and adjusted by a physical therapist who conducted all KT applications in all experimental trials (Lines 239-241).*

*For the bias of inter-rater assessment, we meant to present the information that, since we had only one physical therapist perform all KT taping, there would be no bias between inter-rater. To avoid confusion, we now have removed this sentence.*

• It still not clear how did you determine the 100KT tension without “fracturing” the tape and compromising its elastic properties. This “pre-experiment” to determine the tension should be detailed, at least within a supplement of the paper. If you applied a length-tension curve and determined the maximum tension, the so-called 100% would be the fracturing point? Hence, the 100KT is not actually the maximum tension, whereas it would make more sense to use the greatest tension before reaching the fracture point (you could call that 100KT for the purpose of your experiment, but this information needs clarification). In addition, what was the reliability within this “pre-experiment”?

*Response: Thank you for bringing this concern, and we agree your point. In detail, during the “pre-experiment”, the physical therapist stretched the KT to* *the point where a strong resistance could be felt (passing this point would lead to the fracture of the tape). Indeed, 100KT would be the point where the tape breaks. Thus, we have revised across the entire manuscript, instead of calling “0KT, 50KT, and 100KT”, we now call them “no tension (0KT), moderate stretch KT (ModKT), and maximal stretch of KT (MaxKT)”. We also added a sentence for clarification (Lines 248-249): “ tape stretch tension was measured with increment of stretch length every 0.5 cm until the maximal stretch intensity (the point where a strong resistance could be felt, thus passing this point could lead to the fracture of the tape).”*

*The raw data in the pre-experiment we only used a single KT strip to test the length-tension relationship. Each length condition (from 16 cm to 23.5 cm) was tested 5 times in a single measure. Therefore, we didn’t perform intra- and inter-reliability for the pre-experiment.*

• I still do not agree with the inclusion of the VAS measurement. From my standpoint, this is a subjective measurement and the focus of your study is the h-reflex measurement. By including the VAS you are dealing with another topic that was not covered by your study rationale and does not add to your research question. My recommendation is to erase this data, as this information does not add to the clinical practice, instead, this might bias the reader as they might wrongly assume that a “feeling” of tension could represent an effect. This is not the case and you should refrain from subjective measurements at this point. The h-reflex might be of more value if you successfully “translate” the clinical and practical findings provided by your study.

*Response: Thank you for bringing this out again. We agree with you that the VAS information should not be a part of this paper. We therefore removed the relevant information from the Methods, Results, and Discussion sections.*

RESULTS
• Overall, the results section improved.
• In table 1, perhaps my suggestion was not clear in the previous review: please include the “raw” data (mean and SD), for each variable on each condition (0KT, 50KT and 100KT) stratified by lying and standing. Besides the “raw” data, then include the mean difference together with the CI95% for each condition. This is an adequate presentation of your results, which my help the reader and other researchers to interpret your findings. Moreover, by including this detail on Table 1, I suggest removing Fig 4.

*Response: Thank you for the further clarification. Now we have revised Table 1 based on your comments. In addition, Figure 4 is removed.*

DISCUSSION
• Lines 364-378: As I have suggested, please remove the VAS findings and this discussion, which does not add to your research question and might bias the reader. I suggest focusing on neuronal excitability and posture variations.

*Response: Thank you. We have removed the VAS findings according to your comments. And We have also structured the Discussion to make it focus on neuronal excitability and posture variations.*

• Line 393: The other studies included in the introduction should also be considered in the discussion. This might put your data into context by comparing the studies with your data. Interestingly, even though the tensions were different between studies, most of them have similar findings compared to your study (KT does not influence motoneuronal excitability). Could you discuss further?

*Response: Thank you for the great suggestion. We have now added these studies to this paragraph. Also, the paragraph was rewritten (Lines 398-425) to discuss the potential reason why KT does not influence motoneuronal excitability.*

• I would suggest a more deepened discussion on why the tape tension did not influence motoneuronal excitability. What might explain your findings? What are the clinical interpretations of your findings?

*Response: Thank you for the suggestion. Our rewritten paragraph (Lines 398-425) provided a possible reason for the nonsignificant finding on H-reflex. In addition, we also added the clinical interpretation of the findings: “with the current KT tape, the tension doesn’t seem to provide meaningful benefits in terms of manipulating spinal motoneuron excitability. Therefore, for clinicians such as physical therapists conducting KT application, tape tension should not be a concern to modulate the sensorimotor activity ascribed to elastic taping.”*

• Please remove the mention to subjective measurements. The discussion mentions quite frequently what the subjects felt, and this should be improved to a more deepened discussion on physiological aspects and underlying assumptions adopted by the KT method, which was not confirmed by your data.

*Response: Thank you. These contents have been taken care of.*
