# Supplementary material for: Acute effects of kinesiology tape tension on soleus muscle h-reflex modulations during lying and standing postures
Source: PLoS One. 2020 Jul 30;15(7):e0236587. doi: 10.1371/journal.pone.0236587 (PMC7392227; doi:10.1371/journal.pone.0236587)
Supplement: Attachment — Submitted filename: Response to Reviewers PONE-D-20-05949R3.docx [file pone.0236587.s005.docx]

Responses to Comments from Reviewers

**Manuscript Number:** PONE-D-20-05949R3

**Title:** “Acute Effects of Kinesiology Tape Tension on Soleus Muscle H-reflex Modulations during Lying and Standing Postures”

*We thank the editor and the reviewer again for reviewing this manuscript. The following are the comments from the reviewer, and we have revised the manuscript based on the suggestions.*

Reviewer #1:

The current version of the manuscript was improved and it is suitable for publication. I have few minor suggestions at this time.

Specific comments:
DISCUSSION
• Lines 423-425: please remove the sentence “Therefore, for clinicians such as physical therapists…”. This sentence does not add, as the previous one is objective enough and shows that tape tension does not provide meaningful effects to motoneuron excitability.

*Response: Thank you. The sentence has been removed.*

• Lines 459-460: The sentence “KT application altered tension perception” should be removed, considering that the VAS data was removed from the manuscript. Please revise the text accordingly and remove any mention to the subjective measurements.

*Response: Thank you. The sentence has been revised to “In conclusion, the current experiment used different tape tensions during KT application on the soleus muscle at both standing and lying postures. The spinal motoneuron excitability…”*

• Lines 466-468: please remove the sentence: “Thus, when applying the KT to individuals, the tension to be used should not be a concern in terms of modulating the sensorimotor activity ascribed to elastic taping”.

*Response: Thank you. The sentence has been removed.*
